# Decreased coronary arteriolar response to K_Ca_ channel opener after cardioplegic arrest in diabetic patients

**DOI:** 10.1007/s11010-017-3264-x

**Published:** 2018-01-05

**Authors:** Yuhong Liu, Victoria Cole, Isabella Lawandy, Afshin Ehsan, Frank W. Sellke, Jun Feng

**Affiliations:** 1 Division of Cardiothoracic Surgery, Cardiovascular Research Center, Rhode Island Hospital, Alpert Medical School of Brown University, Providence, RI USA; 2 Cardiothoracic Surgery Research Laboratory, Rhode Island Hospital, 1 Hoppin Street, Coro West Room 5.229, Providence, RI 02903 USA

**Keywords:** Calcium-activated potassium channels, Cardioplegia, Cardiopulmonary bypass, Diabetes, Coronary microcirculation, Endothelial-dependent hyperpolarizing factor

## Abstract

We have recently found that diabetes is associated with the inactivation of the calcium-activated potassium channels (K_Ca_) in endothelial cells, which may contribute to endothelial dysfunction in diabetic patients at baseline. In the current study, we further investigated the effects of diabetes on coronary arteriolar responses to the small (SK) and intermediate (IK) K_Ca_ opener NS309 in diabetic and non-diabetic patients and correlated that data with the changes in the SK/IK protein expression/distribution in the setting of cardioplegic ischemia and reperfusion (CP) and cardiopulmonary bypass (CPB). Coronary arterioles from the harvested right atrial tissue samples from diabetic and non-diabetic patients (*n* = 8/group) undergoing cardiac surgery were dissected pre- and post-CP/CPB. The in vitro relaxation response of pre-contracted arterioles was examined in the presence of the selective SK/IK opener NS309 (10^−9^–10^−5^ M). The protein expression/localization of K_Ca_ channels in the harvested atrial tissue samples, coronary microvessels, and primary cultured human coronary endothelial cells were assayed by Western blotting and immunohistochemistry. The relaxation response to NS309 post-CP/CPB was significantly decreased in diabetic and non-diabetic groups compared to their pre-CP/CPB responses, respectively (*P* < 0.05). Furthermore, this decrease was greater in the diabetic group than that of the non-diabetic group (*P* < 0.05). There were no significant differences in the total protein expression/distribution of SK/IK in the human myocardium, coronary microvessels or coronary endothelial cells between diabetic and non-diabetic groups or between pre- and post-CP/CPB (*P* > 0.05). Our results suggest that diabetes further inactivates SK/IK channels of coronary microvasculature early after CP/CPB and cardiac surgery. The lack of diabetic changes in SK/IK protein abundances in the setting of CP/CPB suggests that the effect is post-translational. This alteration may contribute to post-operative endothelial dysfunction in the diabetic patients early after CP/CPB and cardiac surgery.

## Introduction

Cardioplegic arrest (CP) in the setting of cardiopulmonary bypass (CPB) is often associated with decreased coronary arteriolar endothelial and vasomotor function [1–10]. In particular, these disturbances are more profound in diabetic patients [10–16]. The mechanisms responsible for CP/CPB-induced coronary and peripheral microvascular dysfunction have been extensively studied by our groups and others [1–5]. We have found that dys-regulation of electrical signaling in the coronary endothelial cells plays a key role in endothelial dysfunction in patients after cardiac surgery. This electrical signaling is mediated by small (SK) and intermediate (IK) conductance calcium-activated-potassium channels (K_Ca_), which are largely responsible for coronary arteriolar relaxation mediated by endothelium-dependent hyperpolarizing factors (EDHF) [17, 20]. We have demonstrated that inactivation of the endothelial SK/IK channels contributes to CP/CPB-induced microvascular endothelial dysfunction early after cardiac surgery [17–19]. Of significance, we found that diabetes is also associated with the inactivation of SK/IK channels, which may contribute to coronary endothelial dysfunction in the diabetic patients at baseline  [20]. Therefore, we hypothesized that diabetes may further cause down-regulation of SK/IK in the setting of CP/CPB and cardiac surgery. In the current study, we investigated the effects of diabetes on coronary arteriolar responses to the selective SK/IK opener NS309 in diabetic and non-diabetic patients and correlated these effects with the changes in the SK/IK protein expression/distribution in the setting of CP/CPB and cardiac surgery.

## Methods

### Human subjects and tissue harvesting

Right atrial tissue samples were harvested from patients undergoing cardiac surgery before (pre-CP/CPB) and after (post-CP/CPB) exposure of the heart to CP and CPB [7, 8, 13, 14, 17]. Harvested tissue samples were immediately frozen in liquid nitrogen (immunoblotting), fixed in 10% formalin for 24 h followed by paraffinization and sectioning into 5-µm slices (immunofluorescent staining), or placed in cold (5–10 °C) Krebs-Henseleit buffer (microvascular studies) [20]. All procedures were approved by the Institutional Review Board (IRB) of Rhode Island Hospital, Alpert Medical School of Brown University, and informed consent was obtained from all enrolled patients. The patients were then divided into the following two groups: (1) non-diabetic patients (ND) with a normal hemoglobin A1c (HgbA1c) and no history or treatment for diabetes; (2) diabetic patients (DM) with a HgbA1c ≥ 8.5 [13, 14].

### Microvessel reactivity

Coronary microvessels (80–150 µm internal diameters, *n* = 8/group) were dissected from harvested atrial tissue samples taken pre- and post-CPB. Microvessel studies were performed in vitro in a pressurized (40 mmHg) no-flow state using video-microscopy as previously described [17–20]. The vessel was pre-contracted with endothelin-1 (10^−8^–10^−7^ M) to achieve 30–40% of baseline diameter. The selective SK/IK inhibitor NS309 (10^−9^–5 × 10^−5^ M) was added to the organ bath and diameter measurements were taken. We have determined previously that the response of human coronary arterioles to NS309 is endothelium dependent [17, 18, 20].

### Immunoblot

The methods for tissue protein purification, Western blotting, and imaging quantification have been described previously [17–20]. Membranes were incubated overnight at 4 °C with primary antibodies against SK3 and SK4 (IK-1) (Alomone Labs Ltd, Jerusalem, Israel). After washing with TBST, membranes were incubated with the appropriate secondary antibody conjugated to horseradish peroxidase. All membranes were also incubated with GAPDH (glyceraldehyde-3-phosphate) or alpha-tubulin (Cell Signaling, Beverly, MA) as loading controls.

### Immunofluorescence microscopy for tissue section

The detailed methods have been described previously [17–20]. After being washed with PBS, tissue sections of atrial appendage were incubated overnight at 4 °C with anti-SK-3 and SK-4(IK) antibodies (Alomone Labs Ltd) and DAPI, and/or anti- smooth muscle α-actin antibody (Cell Signaling, Beverly, MA). The tissue sections were finally mounted with VECTASHIELD Mounting Medium with DAPI (4′,6-diamidino-2-phenylindole) (Vector Laboratories, Burlingame, CA).

### Cell culture and immunofluorescent staining

Human coronary artery endothelial cells (HCAECs, passage 4) harvested from donors (patients) with and without diabetes (Lonza, Walkersville, MD) were cultured and grown in the EGMTM-2 Bullet Kit medium (Lonza) in a humidified incubator with 5% CO_2_ at 37 °C according to the manufacturer’s protocols and our previous work [20]. HCAECs were fixed by using paraformaldehyde (crossing-linking method) for immunofluorescent staining. After being washed with PBS, the fixed cells were incubated overnight at 4 °C, with anti-SK-2 antibody or anti-SK-4(IK) antibody (Alomone Labs Ltd). The cells were finally mounted with VECTASHIELD Mounting Medium with DAPI (4′,6-diamidino-2-phenylindole) (Vector Laboratories, Burlingame, CA).

### Chemicals

Endothelin-1 and NS309 were purchased from Sigma-Aldrich (St. Louis, MO).

### Data analysis

Data are expressed as the mean ± SD. A paired *t* test was performed for the data analysis of patient characteristics. For analysis of categorical data, Fisher’s exact test was employed. One-way ANOVA was employed for protein expression and other imaging. For the analysis of microvessel data, two-way repeated-measures ANOVA with a post hoc Bonferroni test were performed. *P* values less than 0.05 were considered significant. All statistical analysis was performed with GraphPad PRISM-6 Software (GraphPad Software, Inc, San Diego, CA).

## Results

### Patient characteristics

The characteristics of the 16 enrolled patients are shown in Table 1. All patients with pre-operative hypertension were treated with anti-hypertensive medications (*β*-blocker, calcium channel blocker, or angiotensin-converting enzyme inhibitor). The levels of pre-operative blood HgbA1c were 5.1 ± 0.3 in the ND patients, and 9.2 ± 0.4 in the DM patients (*P* = 0.0001).

**Table 1:** Patient characteristics

Patient characteristics	ND	DM	*P* values
Age (years)*	67 ± 8	70 ± 9	0.62
Male/Female (*n*)	5/3	6/2	1.00
HgbA1c (%)*	5.1 ± 0.3	9.2 ± 0.4	0.0001
Patient blood glucose (mg/dL, pre-CP/CPB)*	110 ± 14	216 ± 25 #	0.0001
Patient blood glucose (mg/dL, during CP/CPB)*	145 ± 14	188 ± 12 #	0.0006
Pre-operative insulin (*n*)	0	5#	0.47
Intra-operative insulin (*n*)	1	8#	0.001
Obesity (BMI > 30)	4	5	0.61
Hypercholesterolemia (n)	4	4	1.00
Hypertension (*n*)	7	8	0.3
Atrial fibrillation (*n*)	0	0	1.00
Duration of CPB (min)*	95 ± 16	102 ± 15	0.35
Cross-clamp time (min)*	81 ± 13	86 ± 12	0.41
CABG only (*n*)	5	5	1.00
CABG plus AVR or MVR	3	3	1.00
Oral antidiabetic medications	0	6#	0.002
Aspirin	6	7	0.52

*HgbA1c* hemoglobin A1c, *CABG* coronary artery bypass grafting, *AVR* aortic valve replacement, *MVR* mitral valve replacement, *ND* non-diabetics, *DM* diabetics

* Mean ± SD

# *P* < 0.05 vs. ND

### Microvascular reactivity

**Fig. 1:**
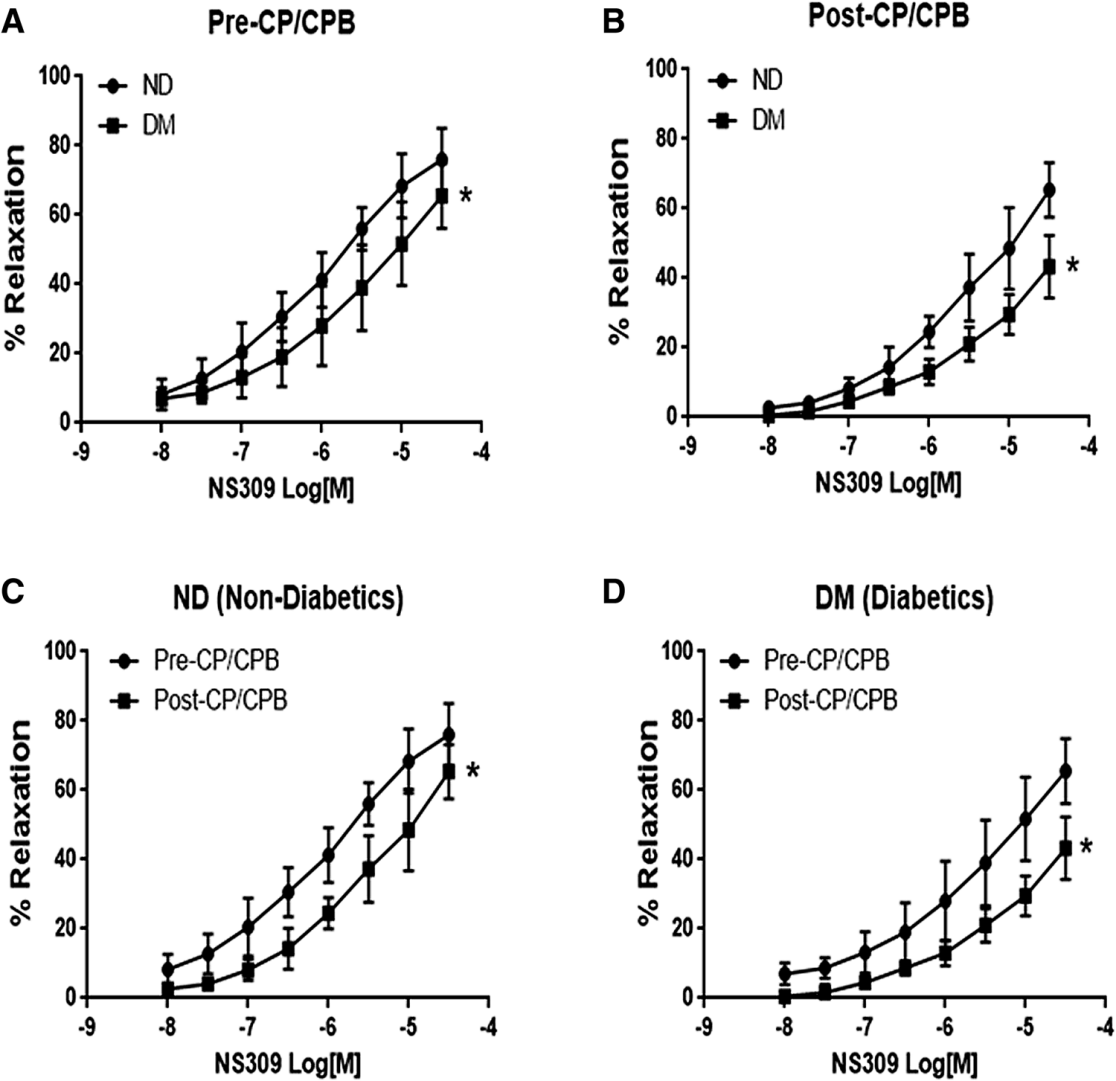
Dose-dependent relaxation response of coronary arterioles to the selective SK/IK activator NS309 in the setting of diabetes (DM) and cardioplegic arrest and cardiopulmonary bypass (CP/CPB). **a** Relaxation response to NS309 in diabetics and non-diabetic patients (ND) at baseline (Pre-CP/CPB); **b** relaxation response to NS309 Post-CP/CPB in ND and DM; **c** relaxation response of ND microvessels to NS309 between Pre- and Post-CP/CPB; **d** relaxation response of DM microvessels to NS309 between Pre- and Post-CP/CPB; Mean ± SD, *n* = 8/group. *P < 0.05 versus ND or Pre-CP/CPB;

The selective SK/IK inhibitor NS309 caused a dose-dependent relaxation response in both ND and DM microvessels (Fig. 1). At baseline (pre-CP/CPB), the relaxation response of coronary arterioles to the selective SK/IK inhibitor NS309 was significantly decreased in the DM group compared to the ND group (Fig. 1a, *P* < 0.05). The post-CP/CPB responses to NS309 were significantly diminished in both ND and DM groups (*P* < 0.05 vs. their pre-CPB, Fig. 1c, d), but the reduction was greater in the DM group than that of the ND group (*P* < 0.05, respectively, Fig. 1b).

### Effect of CP/CPB on levels of SK-3 and SK-4(IK-1)

**Fig. 2:**
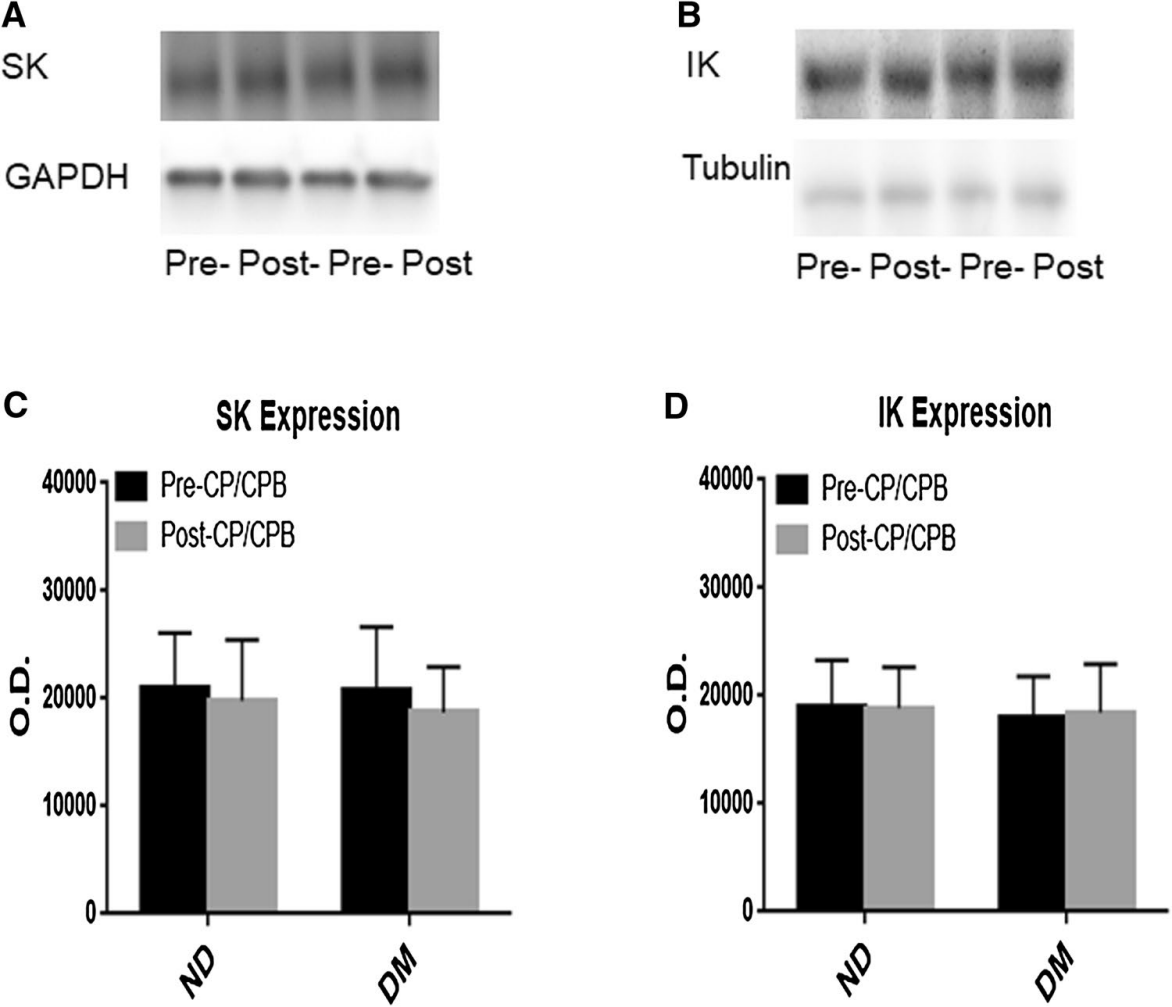
**a, c** Representative immunoblots of human atrial tissue samples harvested pre- and post-CP/CPB from diabetic (DM) and non-diabetic (ND) patients, *Pre-* pre-CP/CPB, *Post-* post-CP/CPB; **b, d** densitometric evaluation of immunoblot band intensity shows no significant differences in the levels of SK-3 (*P* = 0.08 vs. ND) and SK-4 (IK-1) (*P* = 0.3) polypeptides between ND and DM groups or between pre- and post-CP/CPB. Mean ± SD, *n* = 6/group

At baseline (pre-CP/CPB), there were no significant differences in the protein expression of total SK-3 (Fig. 2a, c) and SK-4(IK-1) (Fig. 2b, d) in the atrial myocardium between non-diabetic and diabetic patients (*P* > 0.05). There were no significant changes in SK-3 and SK-4(IK-1) post-CP/CPB compared with their pre-CP/CPB values (*P* < 0.05) and also no significant differences between ND and DM post-CPB (Fig. 2a–d).

### Vascular distribution of SK-3 and SK-4 (IK-1)

**Fig. 3:**
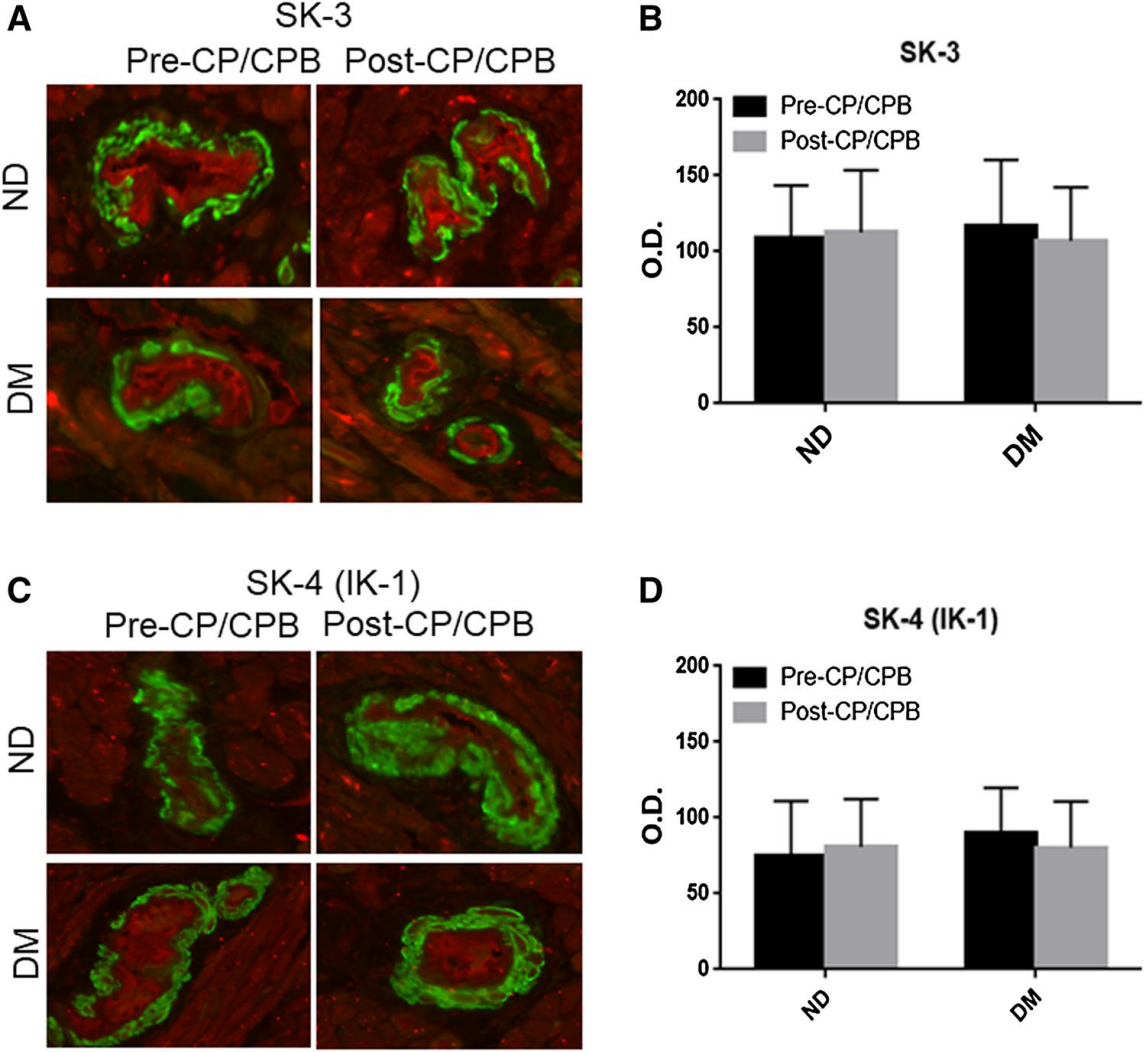
**a, c** Immunofluorescence staining of SK-3 and SK-4 (IK-1) in paraffin-embedded human atrial tissue samples from ND and DM patients in the setting of CP/CPB; Vessels were co-stained for smooth muscle α-actin (green), SK-3 or SK-4 (IK-1) red in endothelium; **b, d** densitometric analysis of signal intensities shows no significant differences in SK-3 and SK-4 (IK-1) distribution between the two groups or between pre- and post-CPB; **c** data are means ± SD, *n* = 6/group. (Color figure online)

Immunofluorescent staining of SK-3 and SK-4 (IK-1) was observed in the coronary arteriolar endothelial cells (red, Fig. 3a, c) in atrial tissue slides. There were no significant differences in the immunofluorescent intensity of SK-3 and SK-4 (IK-1) at baseline (pre-CP/CPB) between ND and DM vessels (Fig. 3b, d). The pre-CP/CPB SK-3 and SK-4 (IK-1) were not significantly altered post-CP/CPB in both ND and DM groups (Fig. 3b, d). There were no significant differences in the post-CP/CPB SK-3 and SK-4 immunofluorescent intensity in vessels between ND and DM (*P* > 0.05).

### Endothelial distribution of SK-2 and SK-4 (IK-1)

**Fig. 4:**
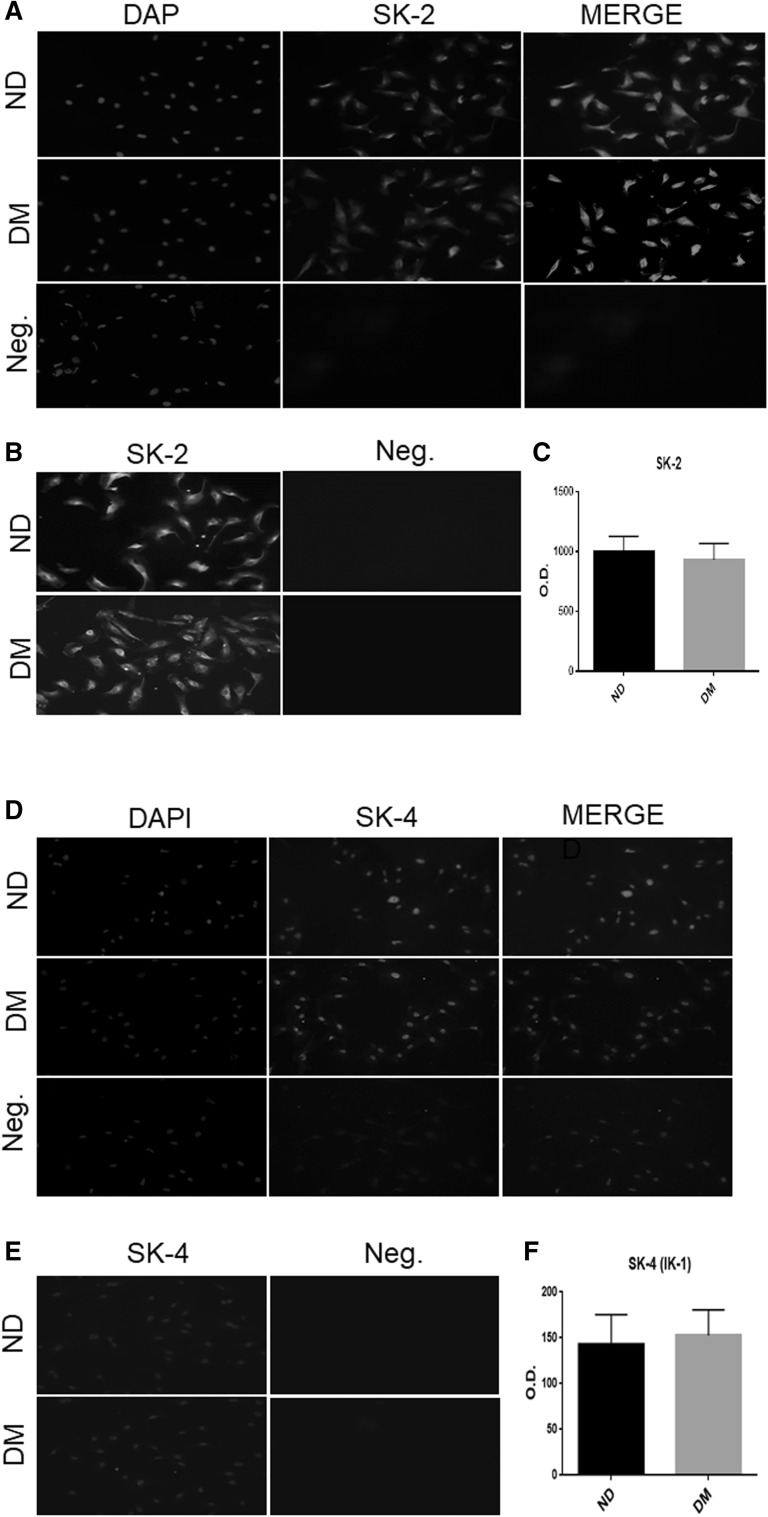
**a, d** Immunofluorescence staining of SK-2 and SK-4 (IK-1) in the cultured human coronary arterial endothelial cells (HCAECs) from ND and DM patients; Cells were co-stained for cell nuclei (blue), and SK-2 (red, **a**) or SK-4 (IK-1) (green, **d**); **b, e** Intensity of SK-2 (**b**) and SK-4 (IK-1, **e**); **c, f** Densitometric analysis of signal intensities shows no differences in SK-2 (**c**) and SK-4 (IK-1, **f**) distribution in HCAECs between ND and DM group Mean ± SD, *n* = 5/group. (Color figure online)

To further test if SK-2 and SK-4 (IK-1) are localized in the endothelium, we performed immunofluorescent staining of SK-2 and SK-4 (IK-1) in the cultured HCAECs. The signals of SK-2 (Red, Fig. 4a) and SK-4 (green, Fig. 4d) were strong in the HCAECs in both ND and DM groups. There were no significant differences in the fluorescent intensity of SK-2 and SK-4 of HCAECs between the ND and DM groups (Fig. 4b, c, e, f, *P* > 0.05).

## Discussion

The present study confirmed our previous findings that CP/CPB is associated with a decreased relaxation response of human coronary arterioles to the selective SK/IK activator NS309, suggesting that CP/CPB is associated the inactivation of endothelial SK/IK channels [17, 18]. Diabetes is associated with the reduction in endothelial SK/IK current density, endothelial hyperpolarization and SK/IK activator-induced endothelium-dependent coronary arteriolar relaxation in animals and humans [20]. Consistent with a previous study [20], we also observed that the dose-dependent relaxation response to the selective SK/IK activator NS309 was significantly decreased in the diabetic coronary arterioles compared to that of case-matched non-diabetics at baseline (pre-CP/CPB). These findings support the notion that diabetic inactivation of SK/IK contributes to coronary microvascular endothelial dysfunction. Importantly, the current study further demonstrates that diabetes, when combined with CP/CPB, further decreases the relaxation response of coronary arterioles to NS309. This novel finding suggests that diabetes is associated with further inhibition of SK/IK channels in the human coronary microvasculature early after CP/CPB and cardiac surgery. Thus, diabetic inhibition of SK/IK may also contribute to post-operative endothelial dysfunction in the diabetic patients early after CP/CPB and cardiac surgery.

Consistent with our previous study, we found that SK/IK channels are predominately present in the endothelial cells of human coronary microvessels and that CP/CPB did not change the total protein expression/localization of SK/IK in the non-diabetic patients [17, 18]. Diabetes-induced changes in endothelial SK/IK function and expression have been studied in detail in diabetic rodents [21–24]. For instance, decreased or increased mRNA/protein expression of endothelial SK/IK channels underlies the impaired EDHF-dilator response in the conduit artery of rodents [22–24]. In contrast, we recently observed that diabetes did not alter the expression/distribution of SK/IK polypeptides in the human myocardium or coronary microvessels at baseline (pre-CP/CPB) [20]. The current study confirms our previous findings by showing no changes in the total SK/IK protein expression/distribution in the diabetic heart and coronary vasculature at baseline. In addition, our study further indicates no alteration in SK/IK distribution in the diabetic HCAECs at baseline. To further examine if diabetes affects SK/IK protein expression/localization in the human myocardium and microvasculature in the setting of CP/CPB and cardiac surgery, we performed immunoblot and immunohistochemistry to quantify and localize SK/IK channel polypeptides. Notably, we found that diabetes did not affect SK/IK protein expression/distribution in the ischemic myocardium and coronary arterioles early after CP/CPB and cardiac surgery. The lack of diabetic changes in SK/IK protein levels in the setting CP/CPB suggests that the effect is post-translational.

There are still a number of limitations to the current study. For example, the precise mechanisms responsible for diabetic dys-regulation of SK/IK and endothelial function are still undefined in the current study. Interestingly, our previous work, along with others, have demonstrated that diabetes increases oxidative stress, NADPH oxidase (Nox) and PKC expression/activation in human myocardium, coronary microvessels and endothelial cells in the setting of CP/CPB [12, 13, 25, 26]. Nox and dysfunctional mitochondria mutually stimulate to enhance ROS production, and PKC expression/activation may play a pivotal role in endothelial dysfunction in diabetes and cardiac surgery [12, 13, 25–27]. The persistent overproduction/activation of NADH-Nox, mROS, and PKC in diabetes and cardiac surgery may negatively regulate SK/IK, endothelial function, and coronary arteriolar relaxation. Therefore, future work should focus on how metabolic changes from diabetes regulate SK/IK channels and coronary endothelial function in the setting of CP/CPB.

In conclusion, diabetes further inactivates SK/IK channels of coronary microvasculature early after CP/CPB and cardiac surgery. This alteration may contribute to post-operative endothelial dysfunction in diabetic patients after CP/CPB and cardiac surgery.
